# PLL-Based Readout Circuit for SiC-MOS Capacitor Hydrogen Sensors in Industrial Environments

**DOI:** 10.3390/s22041462

**Published:** 2022-02-14

**Authors:** Andrei Enache, Florin Draghici, Florin Mitu, Razvan Pascu, Gheorghe Pristavu, Mihaela Pantazica, Gheorghe Brezeanu

**Affiliations:** 1 Faculty of Electronics, Telecommunications and Information Technology, University Politehnica of Bucharest, 061071 Bucharest, Romania; andrei.enache1512@upb.ro (A.E.); florin.mitu@upb.ro (F.M.); mihaela.pantazica@cetti.ro (M.P.); 2 National Institute for Research and Development in Microtechnologies (IMT), 077190 Voluntari, Romania; 3 Romanian Young Academy, Research Institute of the University of Bucharest, University of Bucharest, 030018 Bucharest, Romania

**Keywords:** hydrogen detection, gas sensor, phase locked loop, nonlinear capacitor sensor, silicon carbide, MOS capacitor

## Abstract

For proper operation in real industrial conditions, gas sensors require readout circuits which offer accuracy, noise robustness, energy efficiency and portability. We present an innovative, dedicated readout circuit with a phase locked loop (PLL) architecture for SiC-MOS capacitor sensors. A hydrogen detection system using this circuit is designed, simulated, implemented and tested. The PLL converts the MOS nonlinear small-signal capacitance (affected by hydrogen) into an output voltage proportional to the detected gas concentration. Thus, the MOS sensing element is part of the PLL’s voltage-controlled oscillator. This block effectively provides a small AC signal (around 70 mV at 1 MHz) for the sensor and acquires its response. The correct operation of the proposed readout circuit is validated by simulations and experiments. Hydrogen measurements are performed for concentrations up to 1600 ppm. The PLL output exhibited voltage variations close to those discernable from experimental C-V curves, acquired with a semiconductor characterization system, for all investigated MOS sensor samples.

## 1. Introduction

The applications of gas sensors have broadened considerably over time, extending from monitoring concentrations under the olfactory human limit to detecting the presence of dangerous compounds and ensuring work safety. Notably, volatile organic compounds (VOCs), H_2_S and NO_2_ are among the substances of interest when checking whether an environment is habitable or work suitable. In industry, the monitoring of flammable or explosive gases has become essential for guaranteeing fast responses to leaks and avoiding casualties/infrastructure damage [1]. In the search for clean and renewable energy, hydrogen (H_2_) has emerged as one of the leading candidates [2,3,4,5,6,7,8,9,10]. However, it is a highly explosive gas, if its concentration in air exceeds 4.65% [11,12]. Therefore, the need for high performance sensors (and associated readout circuits) to detect H_2_ in multiple applications is increasing rapidly [13]. Oftentimes, these applications are in harsh environments, such as the aerospace domain [11,14,15] the industrial sector [16,17] or, more recently, the automotive field for hydrogen powered vehicles [18,19].

Among detectors with various transduction mechanisms, gas sensors with capacitive components, such as metal-oxide-semiconductor (MOS) capacitors, interdigitated electrode (IDE) capacitors and quartz crystal microbalances (QCM) resonators are preferable due to their size, low cost and sensitivity [20]. MOS structures, in particular, can be fabricated using simple, well-established technological processes and, as opposed to their IDE and QCM counterparts, they do not require special coatings for compound detection [20]. Additionally, their capacitance can also be voltage-controlled, which gives flexibility in tuning a sensor’s baseline output (in an inert atmosphere). Although their optimal operation usually requires heating, this aspect is lower for industrial applications where the various processes that demand gas monitoring also entail elevated temperatures. For such hostile environments, silicon carbide (SiC) H_2_ MOS capacitor sensors are a suitable variant [16,20,21,22,23,24,25]. These sensors have been shown to be able to operate at high temperatures [16,20] up to 500 °C [16]. They also exhibit short response and recovery times [20] and good sensitivity to even small concentrations of H_2_ (as low as 20 ppm [22]). This optimal behavior is achieved with the MOS capacitor biased in the region where the capacitance is strongly dependent on applied voltage. In most prior studies [16,20,21,22,23,24,25] these performances were measured under laboratory conditions, with the sensor small-signal C-V characteristics extracted using semiconductor parameter analyzers. In order to ensure precision, noise suppression, energy efficiency and portability requirements for sensor operation in real conditions, a readout circuit is mandatory [26,27,28,29,30]. However, contributions pertaining to such circuit architectures suitable for SiC-MOS capacitor hydrogen sensors are scarce [30].

The topology described in [30] used a quad-diode bridge circuit with the MOS capacitor sensor connected as the bridge load. In this architecture, a variation in the active sensor capacitance led to a shift in the common mode DC voltage at the bridge input.

This paper proposes a portable hydrogen detection system with alternate readout circuit architecture. The sensing element is a SiC-MOS capacitor. The innovative readout circuit is essentially a phase locked loop (PLL) [31,32,33,34].

Readout circuits with PLLs have been previously used for gas sensing [35,36,37]. In these applications, the effective PLL block performs modulation/demodulation, enabling accurate frequency sweeping (necessary for sensing with high specificity and sensitivity). The main drawback is the high operating frequency (over 200 GHz), requiring implementation in advanced SiGe processes [35,36,37].

For our work, the PLL readout circuit was used as a capacitance to voltage converter, at a relatively low operating frequency (1 MHz). Its purpose was to reproduce the measurement conditions given by the parameter analyzer, but in a cost-effective form (for use in industrial applications). As the sensor was exposed to hydrogen, its C-V characteristic shifted [22] and the PLL varied the bias to maintain a constant capacitance, thus generating a voltage value proportional to the H_2_ concentration. Moreover, the PLL topology allowed for selecting the initial sensor bias voltage (i.e., at 0 ppm H_2_) to achieve optimal sensitivity [33]. Unlike conventional readout circuit architectures [30] it does not require dedicated bias and amplification blocks.

Section 2 describes the technological process flows for the SiC-MOS capacitor sensor, the structure of the PLL architecture and constituent blocks’ design. Section 3 presents simulation and experimental results demonstrating the correct operation of the proposed PLL topology. H_2_ measurements were carried out to validate the functionality of the complete hydrogen detection system.

## 2. Materials and Methods

The proposed system comprises two main parts: a SiC-MOS capacitor hydrogen sensor and a PLL-based readout circuit, designed for implementation with discrete components. The system generates an output voltage proportional to the hydrogen concentration detected by the sensor.

### 2.1. Hydrogen Sensor Structure

The SiC-MOS capacitors were fabricated starting from a 4H-SiC wafer with epitaxial layer. The doping concentration of the epi layer is around 2 × 10^16^ cm^−3^, while the substrate is heavily doped (−10^18^ cm^−3^).

**Figure 1:**
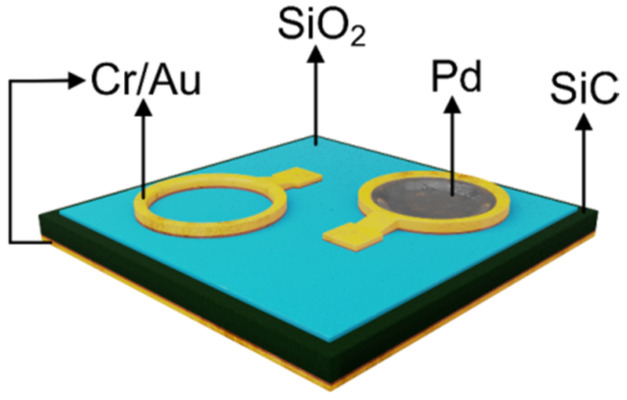
SiC MOS hydrogen sensor structure.

A top view image of the structure is depicted in Figure 1. A 1 µm thick field oxide was deposited through low pressure chemical vapor deposition on the front side of the SiC surface. The active areas (400 µm diameter circular windows) were defined by etching with a ramp oxide profile [20]. The MOS oxide (SiO_2_) was grown in a dry oxygen atmosphere at a temperature of 1100 °C for 4 h, resulting in a thickness of 33 nm. After the oxidation process, a rapid postoxidation annealing was performed in a N_2_ atmosphere, at 1100 °C for 6 min. This treatment was demonstrated to both diminish the interface states density and improve the performance of the MOS device as hydrogen sensor [22]. Subsequently, a layer of Pd (50 nm) was deposited and defined on the window (Figure 1). Pd is a well-known catalytic metal with high hydrogen solubility [16]. The backside ohmic contact and the front side pads were formed by a successive deposition of Cr (15 nm)/Au (100 nm). The final devices were diced into chips and encapsulated in TO 39 packages using wire bonding technology.

The supplementary contact pad shown in Figure 1 is a “dummy” structure used for parasitic capacitance evaluation [20].

### 2.2. Hydrogen Detection Principle

Several interactions between hydrogen and our SiC-MOS structures were responsible for the overall sensing mechanism [22]. First, as molecular H_2_ was adsorbed at the active sensor area, it was dissociated into atomic form H^+^ at the Pd/SiO_2_ interface. As such, the Pd work function chemically shifted. Cumulatively with this effect, charged states presented at the metal-oxide interface were also passivated, creating a dipole layer between Pd and SiO_2_. However, it was shown that the main contributor to hydrogen sensitivity was given by the further diffusion of H^+^ deeper into the oxide and interaction with SiO_2_ bulk traps [22,38,39,40]. The trade-offs for this increase in susceptibility to H_2_ were that the structure took a longer time to both stabilize its response and to release stored hydrogen atoms as the environment becomes inert.

**Figure 2:**
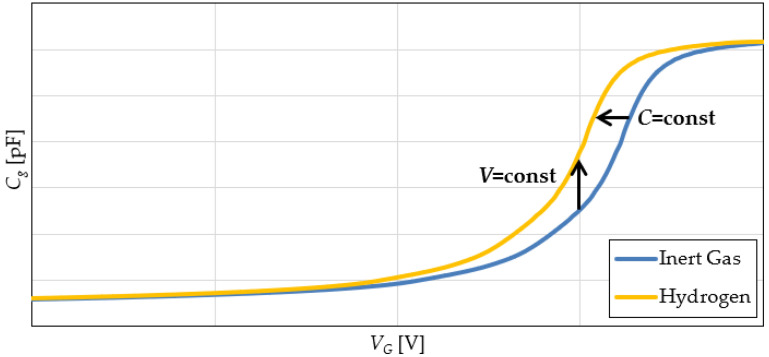
MOS sensor C-V theoretical characteristics (in inert gas and with H_2_ stimulus).

The characteristic of interest for the MOS capacitor sensor was the small-signal capacitance, denoted by *C_g_*, versus the DC bias voltage, *V_G_* (C-V characteristic), measured at a frequency of 1 MHz. Two theoretical C-V curves (in an inert gas and in the presence of H_2_) are given in Figure 2 [22,33]. It can be seen that the presence of H_2_ leads to a left shift of the C-V characteristic [22]. This behavior is most obvious in the region where the capacitance is strongly dependent on the bias voltage. As a result, two hydrogen detection principles may be used, illustrated in Figure 2 [33].

Principle *V* = const implies maintaining a constant bias voltage across the MOS sensor and allowing its capacitance to vary. The most simple transducer in this scenario could be an oscillator which includes the capacitive sensor. Thus, the output quantity would be the oscillator frequency, which would change as the small-signal capacitance varies.

The *C* = const technique involves a constant sensor capacitance, which must be maintained by adjusting the DC bias voltage. In this case, the sensor can again be included in an oscillator, with a carefully chosen structure to allow for sensor bias control. This voltage controlled oscillator (VCO) will then be integrated into a phase locked loop (PLL) [31,32]. The PLL will adjust the bias voltage in order to maintain a constant VCO frequency (and thus sensor capacitance). The output quantity in this case is the sensor bias voltage. It is also the PLL error voltage [31,32].

In our implementation, the *C* = const approach is proposed for two reasons:

- The frequency of the small signal applied to the sensor is constant and can be made equal to the characterization frequency. This is preferable because the sensor capacitance also depends on frequency, and frequency shifts may affect precision;
- In a portable circuit solution, it is easier to process a constant voltage output than a frequency output.

### 2.3. Readout Circuit

#### 2.3.1. Phase Locked Loop

**Figure 3:**
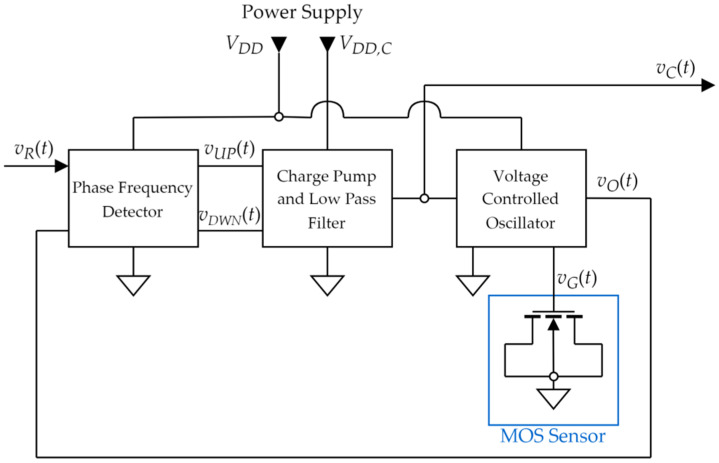
Proposed Digital PLL readout block schematic.

The proposed readout circuit is a digital phase locked loop (PLL) structure [31] intended to operate at a frequency of 1 MHz (the characterization frequency of the sensor). Its block schematic is shown in Figure 3.

The readout circuit architecture in Figure 3 has three main blocks:

- A voltage controlled oscillator (VCO) [33,34] generates an output periodic wave *v_O_*(*t*) with a frequency determined by the control voltage *v_C_*(*t*). It also applies a voltage *v_G_*(*t*) across the sensor, with a DC component (roughly equal to *v_C_*(*t*)) and an AC component. It is critical for accurate H_2_ measurement that the AC component be a small signal (<100 mV peak-to-peak).
- A phase frequency detector (PFD) acquires the phase shift between VCO output *v_O_*(*t*) and a reference input signal *v_R_*(*t*), resulting from their frequency imbalance. It generates pulsed UP and DOWN signals (*v_UP_*(*t*), *v_DWN_*(*t*) Figure 3) with variable duty cycles, indicating whether the control voltage should be increased or decreased;
- A charge pump (CP) and low pass filter (LPF) generate the control voltage based on the duty cycle difference between the UP and DOWN signals. The LPF uses an active inverting structure and has a key role in ensuring PLL stability [31].
- The proposed PLL structure requires two supply voltages:
- *V_DD_*—low voltage supply—for powering the oscillator and the low voltage logic in the phase frequency detector;
- *V_DD,C_*—high voltage supply—for powering the charge pump and active low-pass filter.

In brief, the intended operation of the PLL is based on negative feedback: when the gas concentration increases, MOS sensor capacitance will tend to increase (see Figure 2). This leads to a decrease in the frequency of *v_O_*(*t*) (Figure 3), becoming lower than the frequency of *v_R_*(*t*). The PFD will detect this and set the UP signal to a duty cycle higher than that of the DOWN signal. Therefore, the UP command becomes dominant, and the CP and LPF blocks will decrease the control voltage *v_c_*(*t*). As a result, the bias voltage of the MOS gas sensor is decreased, thus also lowering the capacitance. In this manner, the PLL maintains a VCO frequency equal to the reference frequency, with the consequence being that the sensor capacitance is also kept constant (as per principle *C* = const from Figure 2). The output of the entire sensing ensemble is considered to be the oscillator control voltage (*v_C_*(*t*), Figure 3).

The supply voltages have separate filters for each of the blocks (and sometimes for individual elements of the same block, such as for the CP and the LPF). These filters are designed to ensure at least a 40 dB noise reduction from the supply of one block to the supply of another at the intended operating frequency of 1 MHz.

#### 2.3.2. Voltage Controlled Oscillator

**Figure 4:**
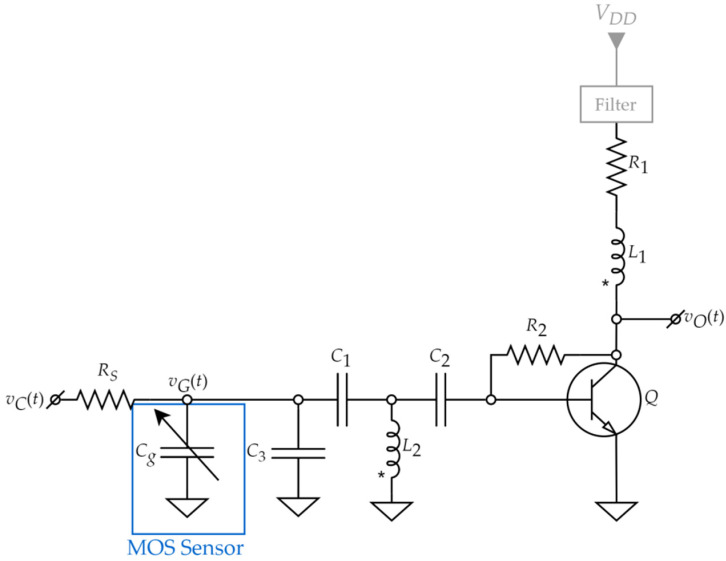
Proposed Armstrong VCO schematic.

The proposed VCO was developed starting from the Armstrong architecture [41] and is shown in Figure 4 [33]. 

In the schematic from Figure 4, the bipolar transistor *Q* is the amplifying element [33]. Its quiescent point is set through resistors *R*_1_ and *R*_2_. The positive feedback loop is created through the coupled inductors *L*_1_ and *L*_2_. The theoretical oscillation frequency is given by [33]:

(1) $$f_{osc}=\frac{1}{2\pi \sqrt{L_{2}\left[\frac{C_{1}\left(C_{3}+C_{g}\right)}{C_{1}+C_{3}+C_{g}}+\frac{C_{2}C_{\mathrm{IN}}}{C_{2}+C_{\mathrm{IN}}}\right]}}$$

where *C_g_* is the sensor capacitance, *C*_3_ is used to trim the oscillation frequency and *C_IN_* is the input capacitance of the transistor. *C*_1_ and *C*_2_ are decoupling capacitors, which should have little effect on *f_osc_* due to their large capacitance. They are needed to separate the DC bias circuit of the transistor, the *L*_2_ inductor (DC voltage is 0) and the sensor voltage (DC voltage equal to *v_C_*(*t*)). For correct decoupling, *C*_1_ and *C*_2_ should be selected to be at least an order of magnitude larger than either *C_IN_* or *C*_3_ + *C_g_*. Under these conditions, the oscillation frequency can be approximated as:

(2) $$f_{\mathrm{osc}}=\frac{1}{2\pi \sqrt{L_{2}\left(C_{3}+C_{g}+C_{\mathrm{IN}}\right)}}$$

*C_IN_* is given by

(3) $$C_{\mathrm{IN}}=C_{\pi }+C_{\mu }\left(1+\left|A_{v}\right|\right)$$

In which *Av* is the voltage gain of the amplifier formed with Q, *R*_1_ and *R*_2_.

For high system sensitivity, *C_g_* should be dominant in establishing the oscillation frequency of the VCO. If *C_IN_* is too large, the influence of the sensor capacitance on the oscillation frequency is reduced and detecting small changes in gas concentration becomes difficult. On the other hand, *A_v_* should be large enough to ensure sufficient output (*v_O_*(*t*), Figure 4) amplitude, despite the small signal conditions present at the input. As such, Expressions (2) and (3) suggest a critical requirement for the bipolar transistor: internal capacitances (*C*_π_ and *C*_µ_) as low as possible.

*R_S_* (Figure 4) is a separation resistor which allows the sensor voltage *v_G_*(*t*) to have both a DC and a small signal AC component. Its presence is required since, when the PLL is locked, *v_C_*(*t*) is a purely DC signal provided by the LPF. For correct operation of the PLL, the *R_S_* resistance should be significantly lower than the parasitic parallel resistance of the MOS capacitor sensor but high enough to ensure correct separation. Furthermore, stability must also be taken into account when selecting *R_S_*, since it contributes to determining the loop bandwidth. This is because, as seen in Figure 4, *R_S_*, *C*_3_ and *C_g_* form another low-pass filter (in addition to the block illustrated in Figure 3).

The oscillator is designed to achieve the target frequency of 1 MHz (trimmable via *C*_3_. Figure 4) at a control voltage *v_C_*(*t*) of around 4 V [33]. A small signal level (<100 mV) at the sensor node (*v_G_*(*t*)) is also targeted. Given the previous considerations and the design targets, the VCO component types and values from Table 1 are chosen [33].

The main advantage of this topology is that the sensor is biased with a DC voltage referred to ground. This allows for simpler control circuitry (charge pump, CP, and low-pass filter, LPF), since it only needs to generate one bias voltage. Moreover, the output voltage of the PLL (i.e., the voltage across the sensor) is also referred to ground, which simplifies the measurement. For instance, in a topology where the MOS sensor substrate is not connected to a ground, its body potential could change due to supply voltage variations, temperature, etc. Thus, it cannot be considered a constant reference and would have to be resampled for every individual measurement. Furthermore, in such a topology, the measurement could be further complicated by the fact that the substrate potential also has an AC component. Therefore, the Armstrong architecture, with the sensor body at ground, is more suitable for the proposed PLL measurement system.

Another important advantage of the Armstrong topology in Figure 4 is that, if properly designed, it allows the MOS capacitor to operate at small signal levels (<100 mV peak-to-peak). This is essential for linear operation of the sensor and thus for precise measurements.

#### 2.3.3. Phase Frequency Detector

**Figure 5:**
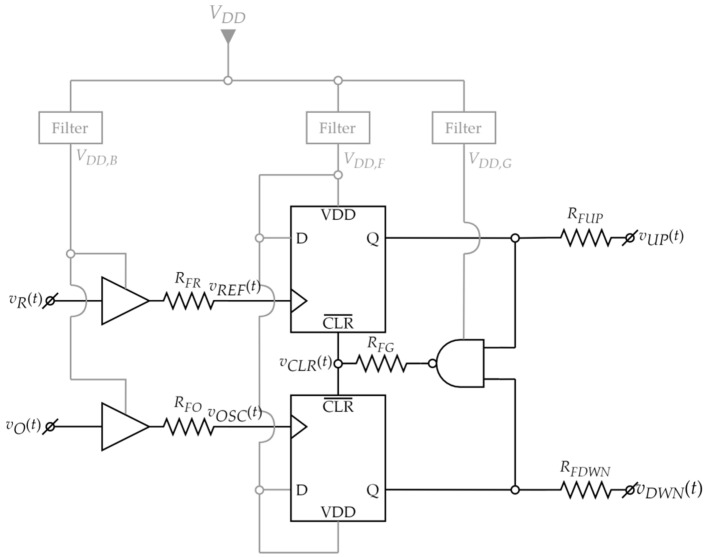
Proposed digital PFD schematic.

The phase frequency detector, based on a well-known digital architecture [31,44] has the schematic shown in Figure 5. The advantage of this topology versus more simple detectors (such as a XOR detector) is that it also processes the frequency difference between the two input signals. Thus, it enables the PLL to lock even if the initial difference between the VCO frequency and the reference frequency is significant [45].

The schematic in Figure 5 comprises:

- Two digital buffers which convert the inputs *v_R_*(*t*) and *v_O_*(*t*) into rectangle wave signals *v_REF_*(*t*) (REF) and *v_OSC_*(*t*) (OSC), respectively;
- Two D flip-flops for generating UP and DOWN signals (*v_UP_*(*t*), *v_DWN_*(*t*));
- A NAND gate to generate the reset *v_CLR_*(*t*) (CLR) signal for the flip-flops;
- Resistors (generically denoted by *R_FX_*) which were added to set the speed of the digital circuits’ outputs by limiting the switching current.

**Figure 6:**
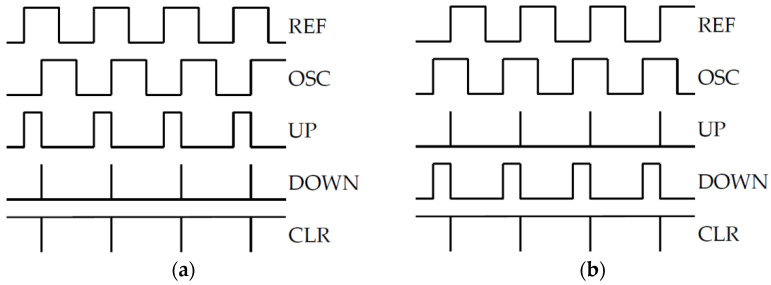
PFD waveforms: (**a**) REF leads OSC; (**b**) OSC leads REF.

The PFD operates as illustrated by the theoretical waveforms in Figure 6 [45]. When *v_R_*(*t*) has a greater frequency than *v_O_*(*t*), the REF rising edges will tend to appear before the ones of the OSC signal (“REF leads OSC”, Figure 6a). Thus, the UP signal goes to logic “1” first. When the rising edge of OSC appears, the DOWN signal also switches to “1”. The NAND gate then detects that both input signals are “1” and generates a short CLR pulse, resetting them both to “0”. Therefore, when REF leads OSC, the UP signal has a greater duty cycle than the DOWN signal. In a similar manner, when OSC leads REF (Figure 6b), the UP signal has a greater duty cycle than the DOWN signal.

In the PLL an increase in the UP-duty cycle drives the VCO to raise its frequency (due to the CP and LPF decreasing the control voltage). For the PLL to lock, the control voltage has to remain constant. Therefore, the PFD has to generate the same UP and DOWN pulses for each oscillation cycle. Consequently, when the loop locks, the VCO signal *v_O_*(*t*) (OSC) will have a constant phase difference versus the reference signal *v_R_*(*t*) (REF). This phase difference is designed to be 0°, as will be explained in Section 2.3.4.

With regard to component choice, first, the digital circuits should be able to operate at low supply voltages in the same domain as the VCO. Ideally, the logic circuits should also be very fast (sharp edges, low propagation delay). However, a compromise needs to be made between switching current and speed. If the maximum switching current is too high, it can create noise on the power supply lines even with filtering.

To modulate the switching current, very fast logic circuits are used, with resistors added in series with their output pins (Figure 5). In this way, for each digital output, the maximum current delivered to the capacitive load of the next stage is limited to *V_DD_*/*R_FX_*, and supply cross talk is reduced.

Note that the addition of resistors *R_FX_* to the PFD architecture yields an improved matching of the switching delays of the two signal paths (*v_r_*(*t*) to *v_UP_*(*t*) and *v_O_*(*t*) to *v_DOWN_*(*t*)). This is because the switching speed is no longer determined by the transistors in the digital circuits, but by the more easily controllable external resistors.

Given the previously mentioned requirements, the component types and values from Table 2 are chosen.

The digital circuits from Table 2 have input capacitances in the order of pF (for instance, SN74LVC74A has a typical value of 5 pF [47]). The devices from the next stage (the charge pump, connected to UP and DOWN signals) will also be selected to have similarly low input capacitance. Therefore, with *R_FX_* set to 1 kΩ, the RC time constants (τ) will be in the order of ns. If switching is considered to be completed after 3τ, then the total delay on each signal path (*v_R_*(*t*) to *v_UP_*(*t*) and *v_O_*(*t*) to *v_DOWN_*(*t*)) is roughly 6τ and thus in the order of tens of ns. These values are at least an order of magnitude lower than the oscillation period (1 µs). Therefore, the proposed PFD implementation can operate correctly at the targeted frequency of 1 MHz.

#### 2.3.4. Charge Pump and Low-Pass Filter

**Figure 7:**
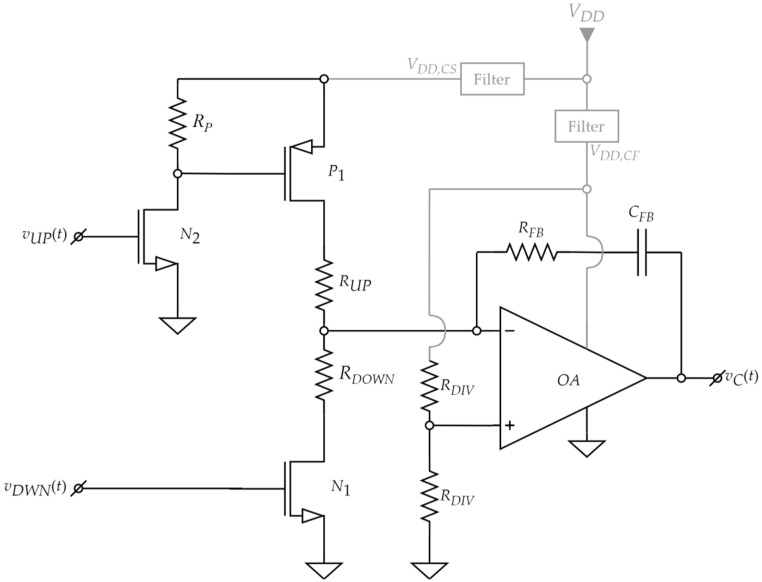
Proposed CP and active LPF schematic.

The charge pump (CP) and the active low-pass Filter (LPF) are designed starting from classical topologies [45]. The proposed schematic comprising both blocks is shown in Figure 7.

The schematic in Figure 7 is powered from the high voltage supply *V_DD,C_* (as shown in Figure 3). This supply voltage must be large enough to ensure the control voltage (*v_C_*(*t*)) range necessary for the MOS hydrogen sensor bias.

The operational amplifier (*OA*) from Figure 7 needs to have a large bandwidth to operate correctly at the targeted PLL frequency of 1 MHz. *OA* is connected in a low-pass configuration [33] with *C_FB_*, *R_FB_* in its negative feedback loop and resistors *R_UP_* and *R_DWN_* at its input. The two identical *R_DIV_* resistors set the input common mode of the amplifier at:

(4) $$v_{–}\left(t\right)≅v_{+}\left(t\right)=\frac{v_{DD,CF}}{2}≅\frac{v_{DD,C}}{2}$$

The role of this LPF is to ensure PLL stability at the imposed operating frequency [31,45] as well as to generate the control voltage *v_C_*(*t*), together with the charge pump.

The CP block requires an inverting level shifter formed with *n*-MOS *N*_2_ and *R_P_*, necessary for driving the gate of the pull-up *p*-MOS *P*_1_ within the 0–*V_DD,C_* range. An acceptable propagation delay for this simple level shifter topology can be achieved if *R_P_* is set at a low enough value. However, this leads to increased power dissipation when *N*_2_ is ON. This effect is mitigated when the PLL locks, if very short “1” pulses are generated on signals *v_UP_*(*t*) and *v_DOWN_*(*t*). Thus, in steady-state operation, the *N*_2_ ON time will be low, significantly reducing average current consumption.

The CP includes the charging stage, formed with pull-up *p*-MOS *P*_1_, pull-down *n*-MOS *N*_1_ and resistors *R_UP_* and *R_DWN_*. Using (4), constant charge (*I_UP_*) and discharge (*I_DWN_*) currents are achieved in the configuration from Figure 7 (when the respective transistors are ON):

(5) $$I_{UP}=\frac{v_{DD,CS}-v_{–}\left(t\right)}{R_{\mathrm{UP}}}≅\frac{v_{\mathrm{DD},C}-\frac{v_{\mathrm{DD},C}}{2}}{R_{\mathrm{UP}}}=\frac{v_{\mathrm{DD},C}}{{2R}_{\mathrm{UP}}}$$

(6) $$I_{\mathrm{DWN}}=\frac{v_{–}\left(t\right)}{R_{\mathrm{DWN}}}≅\frac{v_{\mathrm{DD},C}}{{2R}_{\mathrm{DWN}}}$$

The control voltage variation during a single oscillation cycle (Δ*v_C_*) is given by the change in the voltage across *C_FB_* (see Figure 7) as it is charged/discharged by the constant *I_UP_* or *I_DWN_*, respectively. The active times for these currents, *t_UP_* and *t_DWN_*, are given by the duty cycles of the UP and DOWN pulses (see Figure 6). Thus, Δ*v_C_* can be expressed as:

(7) $$\Delta v_{C}=\frac{I_{\mathrm{DWN}}}{C_{\mathrm{FB}}}t_{\mathrm{DWN}}-\frac{I_{\mathrm{UP}}}{C_{\mathrm{FB}}}t_{\mathrm{UP}}≅\frac{v_{\mathrm{DD},C}}{{2C}_{\mathrm{FB}}}\left(\frac{t_{\mathrm{DWN}}}{R_{\mathrm{DWN}}}-\frac{t_{\mathrm{UP}}}{R_{\mathrm{UP}}}\right)$$

With the proposed VCO, PFD, LPF and CP blocks, the negative feedback operation of the PLL described in Section 2.3.1 is validated because *t_UP_* > *t_DOWN_*, when *v_R_*(*t*) has a greater frequency than *v_O_*(*t*). Therefore, according to Relation (6), the control voltage (the sensor bias) will decrease. Consequently, the sensor capacitance will be lowered (see Figure 2), leading to an increase in the VCO frequency (per Expression (1)). The PLL thus ensures that the VCO frequency follows the reference signal frequency. When the PLL locks onto the reference frequency, the control voltage no longer changes (Δ*v_C_* = 0). Therefore:

(8) $$\Delta v_{C}=0\overset{}{\Leftrightarrow }\frac{t_{\mathrm{DWN}}}{R_{\mathrm{DWN}}}=\frac{t_{\mathrm{UP}}}{R_{\mathrm{UP}}}\overset{}{\Leftrightarrow }\frac{t_{\mathrm{UP}}}{t_{\mathrm{DWN}}}=\frac{R_{\mathrm{UP}}}{R_{\mathrm{DWN}}}$$

Relation (8) shows that when the proposed PLL is locked, the ratio between the duration of the UP and DOWN pulses is constant. If *R_UP_* is chosen equal to *R_DWN_*, the duration of the two pulses will also have to be equal. The PFD structure from Figure 5 cannot generate UP and DOWN pulses of equal duration unless both are very short. This is because as soon as both signals go to logic “1”, the flip-flops are reset to “0” (see also Figure 6). Therefore, to ensure power efficiency, *R_DOWN_* = *R_UP_* is a necessary condition. In this case, the PLL will drive the VCO to generate a signal *v_O_*(*t*) that is in phase with the reference signal *v_R_*(*t*).

Regarding component choice, the transistor switches from Figure 7 must have low input capacitance/gate charge as well as low turn-on and turn-off times. These requirements are similar to those imposed in the PFD design to have a very low PLL loop delay. Furthermore, the *n*-MOS transistors must have a threshold voltage significantly below the chosen *V_DD_* value of 2.7 V, so that *v_UP_*(*t*) and *v_DOWN_*(*t*) signals can drive them into the ON-state. At the same time, they must be able to withstand drain-source voltages equal to *V_DD,C_*.

Considering the previous considerations regarding PLL stability and CP operation, the components from Table 3 are chosen.

## 3. Results

This section presents the measured results for the MOS hydrogen sensors, as well as readout circuit simulations and experimental results for the entire proposed system. Initially, the measurement setup is described.

### 3.1. Measurement Setup

**Figure 8:**
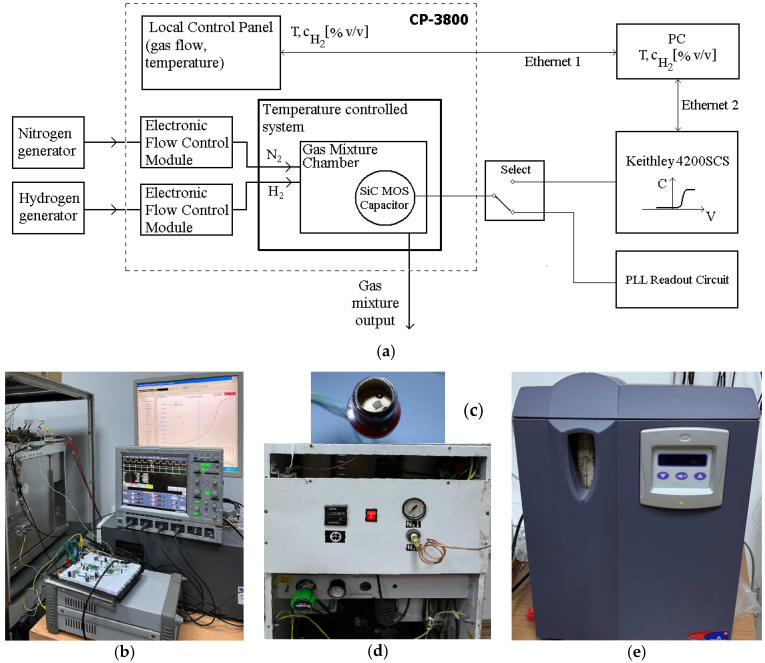
Measurement setup for H_2_ detection: (**a**) block schematic; (**b**) system image; (**c**) SiC MOS capacitor; (**d**) Nitrogen generator; (**e**) Hydrogen generator.

The measurement setup for the hydrogen detection system (block schematic and actual implementation) is depicted in Figure 8. It comprises a Varian CP-3800 chromatograph gas oven (for gas and temperature control) and a Keithley 4200 Semiconductor Characterization System (SCS) for MOS structure bias and C-V measurements (Figure 8b), as well as nitrogen (Figure 8d) and hydrogen (Figure 8e) generators. PLL signals are acquired using a digital oscilloscope (Figure 8b). When the PLL Readout Circuit is connected to the MOS capacitor (Figure 8c), the SCS is decoupled (Figure 8a).

The gases are controlled by mass flow controllers with integrated flow meters. The gas control unit is an adapted version of those used in gas chromatography analytical detectors and can set hydrogen concentrations between 0 and 1600 ppm with steps of 400 ppm.

### 3.2. Experimental Sensor Characteristics

**Figure 9:**
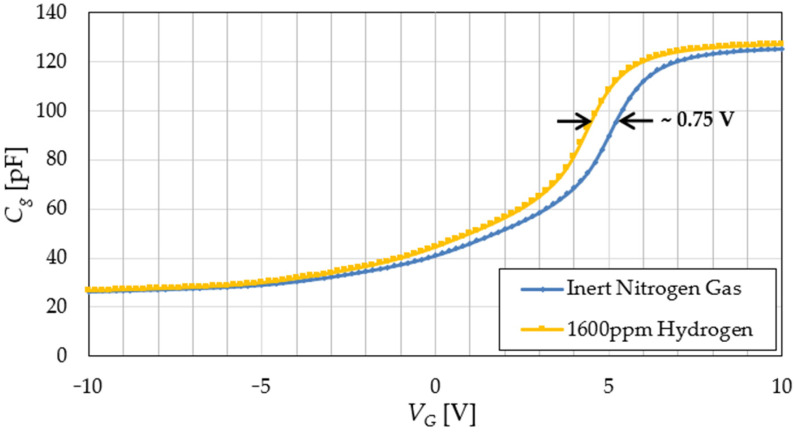
MOS sensor C-V experimental characteristics for sample S1: in inert gas and with 1600 ppm H_2_, respectively.

MOS sensor C-V characteristics were extracted using a Keithley 4200-SCS Parameter Analyzer. The measurements were taken at 1 MHz and 100 °C (higher sensor temperature increases sensitivity [16]). Curves were acquired first in an inert N_2_ atmosphere, then with a 1600 ppm H_2_ concentration. Characteristics for a sample (S1) are shown in Figure 9.

The curves from Figure 9 demonstrate a sensor behavior similar to the one predicted by the theoretical characteristics from Figure 2: as the hydrogen concentration is increased, the C-V plot is variably shifted to the left. Thus, *v_C_*(*t*) = *V_G_* ≅ 5.3 V in inert gas is moved towards approx. 4.55 V at 1600 ppm H_2_ (for *C_g_* = 96.8 pF = const).

Sensor sensitivity (*S*) is defined as this *V_G_* dependence on hydrogen concentration (*c*_H2_):

(9) $$S={\frac{\Delta v_{G}}{\Delta C_{H2}}|}_{C_{g}=\mathrm{const}}$$

Figure 9 shows that good structure sensitivity can be achieved if the PLL biases the sensor with an initial control voltage *v_C_*(*t*) = *V_G_* between 3 and 6 V.

**Figure 10:**
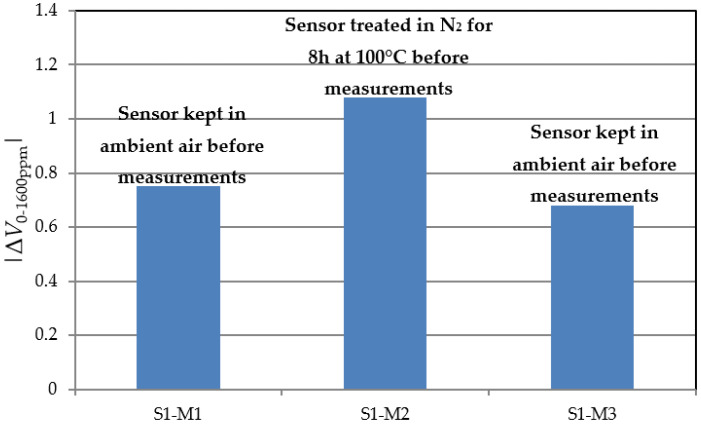
S1 MOS sensor voltage shift after exposure to hydrogen.

Sensor response and, especially, its specificity can be affected by a number of environmental interferences, among which air humidity is the most prominent [20]. To evince this effect for our structures, the S1 sample was characterized in three consecutive days at H_2_ concentrations up to 1600 ppm. Figure 10 presents the SiC MOS capacitor’s voltage shift for each session. The first and third measurement (S1-M1, S1-M3) sets were acquired with the sensor introduced into the test chamber directly from ambient air, while for the second set (S1-M2), the sensor was first kept in the N_2_ atmosphere for 8 h at 100 °C. The baseline sensor bias was tuned for each measurement set to ensure optimal sensitivity (e.g., *V_G_* ≅ 5.3 V for S1-M1, with C-V characteristics depicted in Figure 9).

Figure 10 suggests that exposure to humid environmental air leads the MOS structure to adsorb water vapor, reducing the number of states available for hydrogen detection (S1-M1, S1-M3). Prior treatment of the sensor in a heated atmosphere releases those states and increases H_2_ sensitivity (S1-M2).

### 3.3. PLL Readout Circuit Simulation Results

The proposed VCO structure (the core of the PLL) was previously validated by simulations and experimentally [33]. It was shown to be able to generate a small-signal AC voltage across the sensor (−70 mV peak-to-peak), achieving the design target specified in Section 2.3.1. The focus in this section is on the operation of the PLL system as a whole (Figure 3).

First, the time-domain behavior of the PLL-based circuit was investigated, via transient simulations. Hence, a nonlinear capacitor model was created for the MOS sensor [45] based on the characteristic in inert gas from Figure 9.

**Figure 11:**
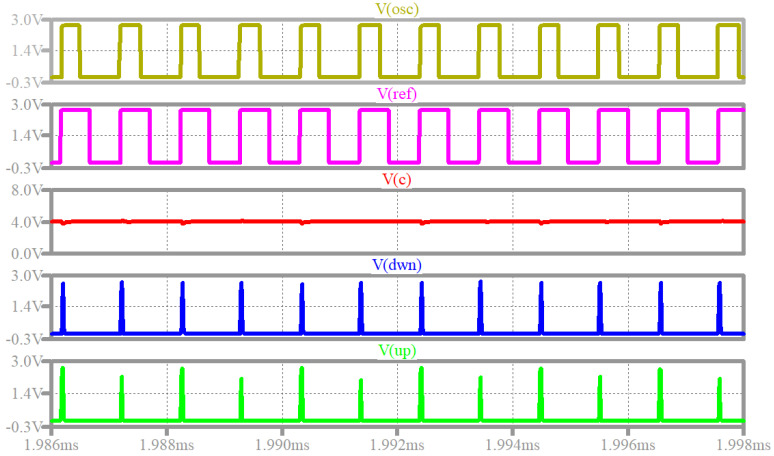
Simulated PLL operation: VCO digital output signal OSC (yellow); digital reference signal REF (magenta); control voltage *v_C_*(*t*) (red); DOWN signal *v_DWN_*(*t*) (blue); UP signal *v_UP_*(*t*) (green).

Figure 11 presents simulated PLL waveforms. The frequency of the reference signal *v_R_*(*t*) (Figure 3) is set to 965 kHz. The control voltage *v_C_*(*t*) (panel 3, in red) is constant, which indicates that the PLL is locked. Another indication of the PLL lock is the fact that the DOWN (panel 4, in blue) and UP (panel 5, in green) signals are nearly identical periodic short “1” pulses, as anticipated in Section 2.3.4. This behavior suggests that the VCO digital output signal OSC (panel 1, in yellow, also, see *v_OSC_*(*t*) in Figure 5) is in phase with the digital reference input signal REF (*v_REF_*(*t*), panel 2, in magenta).

It is important to note that simulations in Figure 11 were carried out with separation resistor *R_S_* set to 4 kΩ. This was required because if a lower value is used, the switching noise seen on the *v_C_*(*t*) signal propagates to *v_G_*(*t*) and also affects the VCO output signal *v_O_*(*t*). Consequently, in simulations the PLL does not lock when *R_S_* is set to 2 kΩ (Table 1). 

**Figure 12:**
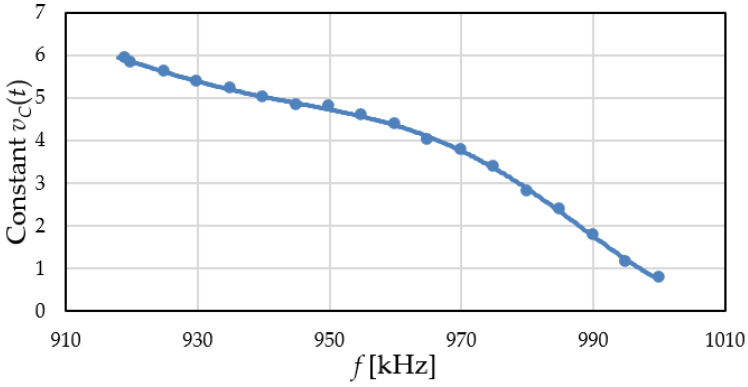
Simulated PLL control voltage vs. reference frequency characteristic.

The simulations from Figure 11 were repeated, varying the input reference signal frequency. The control voltage was evaluated for multiple frequencies when the PLL locks resulting in the constant output voltage *v_C_*(*t*) vs. frequency characteristic from Figure 12.

The shape of the *v_C_*-*f* characteristic from Figure 12 is determined by the nonlinear sensor characteristic from Figure 9 (through the VCO). The possible control voltage range given by the PLL is between 1 and 7 V (limited by the active filter *OA* output range). However, above 6 V, the MOS sensor capacitance variation with bias voltage is greatly reduced (see Figure 8), so the PLL requires long simulation times to lock. For this reason, no data points with *v_C_*(*t*) > 6 V were included. Note that a reference frequency of 965 kHz is associated with a control voltage around 4 V.

The characteristic in Figure 12 illustrates the capability of the proposed circuit to vary the control voltage to match the internal VCO frequency to the reference frequency. This demonstrates its correct operation as a phase locked loop.

### 3.4. Hydrogen Detection System Measurement Results

**Figure 13:**
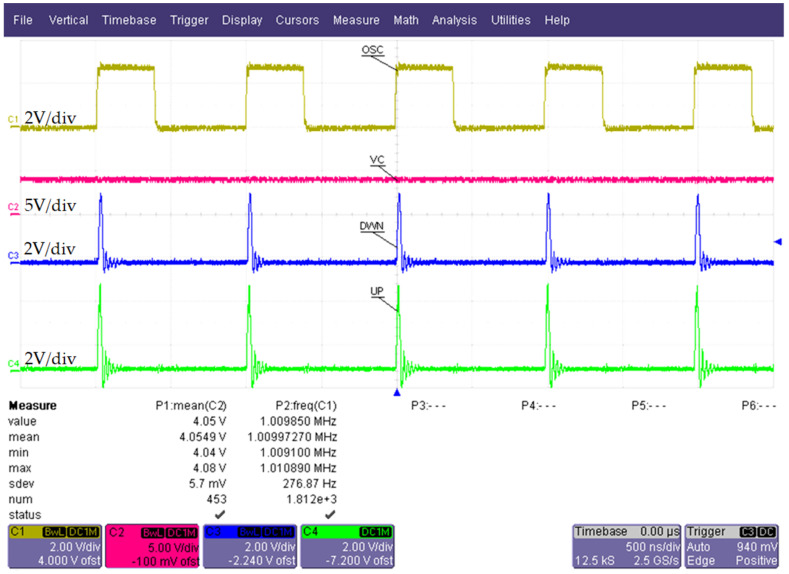
Measured PLL waveforms: C1, OSC signal; C2, control voltage *v_C_*(*t*); C3, DOWN signal *v_DWN_*(*t*); C4, UP signal *v_UP_*(*t*).

As with simulations (Figure 11), the time-domain behavior of the proposed system was confirmed, under lock conditions. Its correct operation is demonstrated by the oscilloscope waveforms represented in Figure 13. The measurement was carried out with no H_2_ stimulus on the MOS sensor and with a *v_R_* frequency *f* ≅ 1.01 MHz.

In Figure 13 the constant control voltage *v_C_*(*t*) (channel 2, in pink) and the short DOWN (channel 3, in blue) and UP (channel 4, in green) pulses indicate that the PLL is locking correctly. Moreover, oscilloscope measurements P1 (*v_C_*(*t*) mean value) and P2 (OSC signal frequency) show that for a control voltage of approximately 4 V, the VCO outputs a center frequency of around 1 MHz, close to simulated results (Figure 11). Thus, the design target described in Section 2.3.2 was achieved experimentally (with the components from Table 1).

Furthermore, the measured average current consumption from the *V_DD,C_* supply (see Figure 3) with locked PLL is roughly 7 mA. This validates the hypothesis that the simple *N*_2_, *R_P_* level shifter from the charge pump (Figure 7) does not lead to increased steady-state power consumption.

It should be noted that the waveforms from Figure 13 were obtained with separation resistor *R_S_* (Figure 4) set to 2 kΩ (as seen in Table 1). When *R_S_* is set to 4 kΩ (shown to work in the simulation), the PLL locks very slowly or not at all. In this scenario, oscilloscope measurements show that *v_C_*(*t*) became less stable (oscillating within −1–2 V of its expected constant value). Thus, the PLL bandwidth decrease due to increasing *R_S_* from 2 kΩ to 4 kΩ is not acceptable experimentally (*R_S_* is part of an RC filter at the VCO input). This suggests that the cutoff frequency of the RC filter is already lower than in the simulation as a result of the equivalent VCO capacitance being larger (due to board parasitics, component tolerances and a higher input capacitance of transistor *Q*, Figure 4).

**Figure 14:**
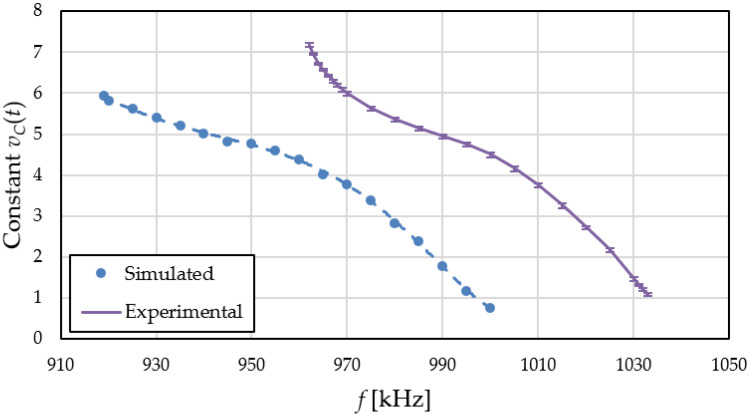
Experimental PLL control voltage vs. reference frequency characteristic alongside simulated data from Figure 10.

The measurements from Figure 13 were repeated, just as with simulations, while varying the input reference signal frequency, with no H_2_ stimulus. The control voltage was measured for multiple frequencies, resulting in the experimental *v_C_*-*f* characteristic from Figure 14. The error bars were obtained by extracting the characteristic 6 times, with the circuit being powered off between measurements.

The experimental *v_C_*-*f* characteristic shows a similar behavior with the simulated one. However, in Figure 14 the entire possible *v_C_*(*t*) range is covered by varying the frequency in a band of −70 kHz (the VCO bandwidth). Looking at Figure 12, the same band is over 80 kHz. Since the MOS sensor capacitance *C_g_*(*V_G_*) is identical in simulations and measurements, the lower experimental bandwidth means that the VCO is less sensitive to its variation. Based on expression (2), this can be attributed to an actual *C*_3_ + *C_IN_* capacitance larger than predicted. This further validates the increased VCO capacitance as the reason for *R_S_* having to be lower in implementation.

The low voltage errors (<100 mV) observed over the entire frequency range (Figure 14) demonstrate the stability and accuracy of the proposed PLL-based readout circuit.

**Figure 15:**
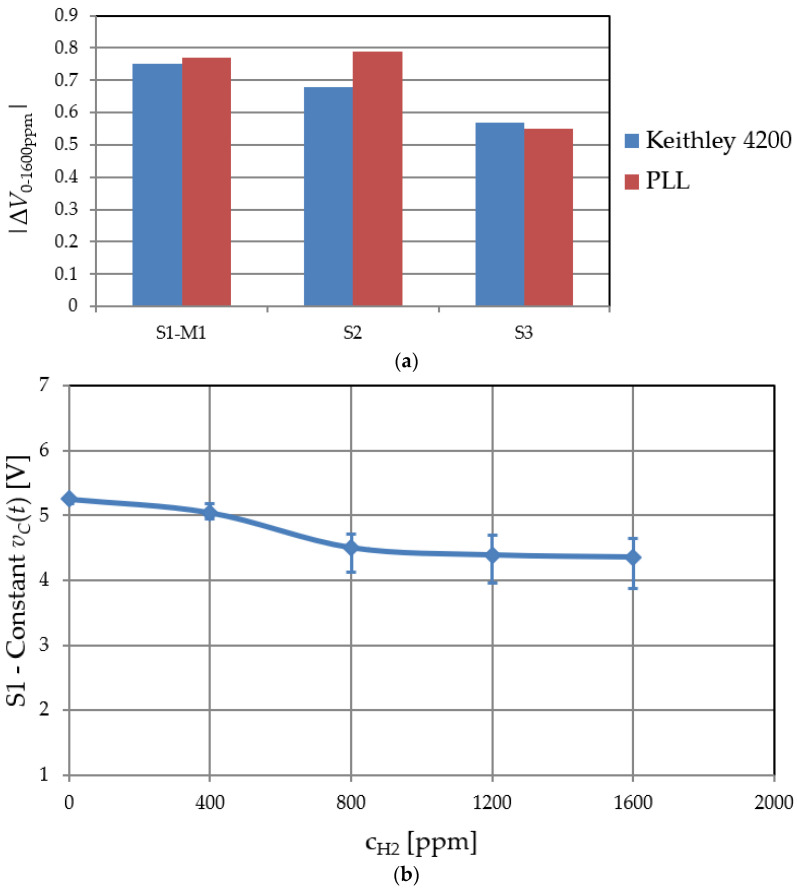
(**a**) PLL output variation vs. SiC MOS capacitor voltage shift (measured with Keithley 4200); (**b**) experimental PLL output voltage vs. hydrogen concentration for sample S1.

Figure 15 shows detection system experiments. The MOS sensors’ voltage variation at ***C_g_* = const** was assessed by both the PLL readout circuit and Keithley 4200 for hydrogen concentrations between 0 and 1600 ppm, increasing every 6 min with a step of 400 ppm. The control voltage was acquired 5 min after setting the concentration to a certain step to allow the generation system to settle. Extending the exposure time past 6 min for a certain concentration would lead to sensor saturation and insensitivity past that point.

Figure 15a illustrates results for three analyzed MOS capacitor structures. For sample S1, the PLL output voltage falls by roughly 0.77 V over the entire H_2_ concentration range. Note that this variation in control voltage is nearly identical to Keithley 4200 measured C-V characteristics corresponding to *C_g_* = 96.8, pF = const. (0.75 V, see Figure 8). Similar agreement was obtained for S2 and S3. Figure 15b details *v_C_* dependence on H_2_ concentration for sample S1 across multiple sets of measurements. The system is sensitive up to 800 ppm H_2_ concentration, only. Past this threshold, the states of the MOS structure become heavily occupied by hydrogen atoms, and sensitivity decreases dramatically.

Thus, the response of the proposed PLL is comparable to the one predicted by the Keithley 4200 MOS sensor measurements, and its correct operation as a readout circuit is validated.

## 4. Conclusions

A hydrogen detection system with a dedicated readout circuit based on a digital phase locked loop topology for SiC-MOS capacitor sensors was designed, simulated, implemented and tested. Readout blocks are essential for industrial applications, ensuring high accuracy, proper signal to noise ratio and portability. The proposed PLL schematic comprises a phase frequency detector, a voltage controlled oscillator and an active low-pass filter with charge pump. The MOS sensor is included within the VCO, and, in the presence of hydrogen, its small-signal capacitance will vary, thus leading to an oscillation frequency shift. This change is detected by the PFD, which through the LPF with CP adjusts the control voltage of the VCO (within the 1–7 V range) to maintain a frequency equal to that of the reference signal. In this manner, the readout circuit generates an output voltage proportional to the H_2_ concentration detected by the sensor. It applies a small AC voltage (−70 mV peak-to-peak) across the MOS sensor, a critical requirement for accurate detection. Moreover, the circuit was shown to reach a VCO frequency equal to the standard C-V characterization frequency of roughly 1 MHz at a control voltage of 4 V, evincing a good agreement between simulations and measurements.

Hydrogen measurements showed PLL control voltage shifts comparable to the variations predicted by the C-V characteristics of all investigated MOS sensor samples. Thus, the correct operation of the proposed PLL-based readout circuit was validated.

The hydrogen detection system’s obtained response variance is attributed mainly to the behavior of the SiC MOS capacitor. Structural optimizations will have to be carried out to improve its resilience to environmental conditions, such as ambient humidity.

## Figures and Tables

**Table 1:** VCO component values and types.

Component	Value/Type
*V_DD_*	2.7 V
*Q*	SS9018 ^(1)^
*R*_1_	2 kΩ ^(2)^
*R*_2_	6.6 kΩ
*L*_1_	37.5 µH ^(3)^
*L*_2_	75 µH ^(3)^
*C*_1_	2.2 nF
*C*_2_	2.2 nF
*C*_3_	100 pF
*R_S_*	2 kΩ

^(1)^ High-frequency transistor, chosen for its low capacitances [42]. ^(2)^ Value used in the implemented PLL, but set to 4 kΩ in simulations (see Section 3.2 and Section 3.3). ^(3)^ Achieved using half of the windings in the transformer PWB-2-CL [43].

**Table 2:** PFD component values and types.

Component	Value/Type
*V_DD_*	2.7 V
Digital buffers	SN74LVC125A ^(1)^
D flip-flops	SN74LVC74A ^(2)^
NAND gate	SN74LVC1G00 ^(3)^
*R_FX_*	1 kΩ

^(1)^ Quadruple bus buffer of which only two are used [46]. ^(2)^ Dual D flip-flops [47]. ^(3)^ Single two-input NAND gate [48].

**Table 3:** CP and LPF component values and types.

Component	Value/Type
*V_DD,C_*	8 V
*N*_1_	FDC6301N ^1^
*N*_2_	FDC6301N ^1^
*P*_1_	FDC6302P ^2^
*OA*	LT1354 ^3^
*R_P_*	50 Ω
*R_UP_*	2 kΩ
*R_DOWN_*	2 kΩ
*R_FB_*	250 Ω
*C_FB_*	2 nF

^1^ *n*-MOS transistor with low input and output capacitances, low switching times [49]; ^2^ *p*-MOS transistor with low input and output capacitances, low switching times [50]; ^3^ Operational amplifier for active filters, 12 MHz gain bandwidth [51].

## Data Availability

Not applicable.
